# Tilt and decentration of posterior and anterior iris-claw intraocular lenses: a pilot study using anterior segment optical coherence tomography

**DOI:** 10.1186/s12886-022-02430-x

**Published:** 2022-05-23

**Authors:** Giacomo Calzetti, Carlo Bellucci, Salvatore Antonio Tedesco, Maurizio Rossi, Stefano Gandolfi, Paolo Mora

**Affiliations:** 1 Ophthalmology Unit, University Hospital of Parma, via Gramsci 14, 43126 Parma, Italy; 2 Institute of Molecular and Clinical Ophthalmology Basel, Basel, Switzerland; 3 Department of Clinical and Experimental Medicine, University Hospital of Parma, Parma, Italy

**Keywords:** CATARACT SURGERY, IRIS-CLAW IOL, TILT, DECENTRATION, ANTERIOR SEGMENT OCT

## Abstract

**Background:**

Information on the centration and tilt of iris-claw intraocular lenses (IC-IOLs) is limited. In this study, we tested the capacity of an anterior segment optical coherence tomography (AS-OCT) instrument to measure decentration and tilt of anterior and posterior IC-IOLs through an integrated software.

**Methods:**

The present observational, cross-sectional study was conducted at University Eye Clinic of Parma (Parma, Italy). The CASIA2 swept-source AS-OCT (Tomey Corp.) was used to measure the tilt and decentration of posterior and anterior IC-IOLs in patients implanted at least 6 months in advance. After failure with full-automation, semi-manual IOL tracing was applied. In-the-bag (IB) contralateral IOLs, when present, were measured automatically. The Bland-Altman method was used to evaluate the agreement between repeated measurements (2 images for each study eye). The amount and direction of tilt and decentration were recorded and plotted into polar charts for evaluation.

**Results:**

A total of 21 patients were included: 14 with posterior and 7 with anterior IC-IOL fixation. In 17 eyes (81%), the AS-OCT provided a repeatable measurement of tilt and decentration. All contralateral eyes with IB IOL were automatically measured. The median decentration was 0.67 mm, 0.24 mm, and 0.24 mm in posterior IC-IOLs, anterior IC-IOLs, and IB IOLs group, respectively. The median tilt was 5.0°, 5.6°, and 5.6° for posterior IC-IOLs, anterior IC-IOLs, and IB IOLs, respectively. Tilt direction was mainly temporal, while decentration was inferior-temporal with posterior IC-IOLs and scattered with anterior IC-IOLs and IB IOLs.

**Conclusions:**

The semi-manual tracing function of the CASIA2 AS-OCT provides repeatable and affordable measurements of the decentration and tilt of IC-IOLs in both the anterior and posterior chamber. Data from the former group were similar to the IB group.

## Background

Since their introduction four decades ago [1], iris-claw intraocular lenses (IC-IOLs) have undergone significant evolution in their design. They are currently used for aphakia correction in the eyes without adequate capsular support due to complicated cataract surgery or lens/bag luxation in pseudoexfoliative syndrome, trauma, and Marfan syndrome. Several studies have explored the safety and efficacy of IC-IOL fixation in the anterior chamber (AC) or posterior chamber (PC) of the eye [2–4]. Although these devices were originally designed to be placed in the AC, retropupillary fixation has become increasingly common, particularly in younger patients. Preservation of the natural lens placement in the PC offers some anatomical and aesthetic advantages. Furthermore, retropupillary fixation may result in lower postoperative corneal endothelial cell loss, although there have been conflicting results in this regard [5–8].

Data on centration and tilt of IC-IOLs is limited because these values are difficult to measure. Different imaging techniques, such as ultrasound biomicroscopy (UBM), Scheimpflug camera, and anterior segment optical coherence tomography (AS-OCT), have been used to assess the positioning of IC-IOLs in aphakic [9–13] or phakic eyes [14–22]. The measurement of IC-IOLs decentration and tilt has been performed mainly in phakic IC-IOLs implanted in the AC [17, 22, 23]. An attempt to evaluate these parameters in IC-IOLs implanted in aphakic eyes was conducted by UBM in a pediatric series [24]. Another study assessed tilt and decentration of scleral fixated IOLs using AS-OCT [25].

The second generation of AS-OCT can be used to measure the tilt and decentration of the IOL with reference to corneal topography [26]. Because the corneal vertex is not affected by the pupil shape, the corneal topographic axis theoretically represents a better reference than the pupil center to assess these parameters, particularly in cases of pupil distortion.

In the present pilot study, we used a second-generation high resolution AS-OCT to measure decentration and tilt of IC-IOLs implanted in the AC or PC of aphakic eyes.

## Methods

This cross-sectional study included patients who received an IC-IOL implant in the AC or PC at the Ophthalmology Unit of the University Hospital of Parma (Parma, Italy). The study protocol was approved by the local Ethics Committee (#552/19), written informed consent was obtained, and all procedures adhered to the tenets of the Declaration of Helsinki. Inclusion criteria were age ≥ 18 years and implantation of an Artisan® (Ophtec, Groningen, The Netherlands) IC-IOL in an aphakic eye 3 years to 6 months prior. Exclusion criteria were corneal opacity preventing slit-lamp visualization of the eye anterior segment, severe perioperative complications (such as iridodialysis, IC-IOL disenclavation, intraocular bleeding, or endophthalmitis), and persistent ocular hypo/hypertension, defined as intraocular pressure ≤ 5 mmHg or ≥ 25 mmHg on at least three measurements in the postoperative medical history. Participants underwent a comprehensive ophthalmological examination including best-corrected visual acuity (BCVA) assessment (by Early Treatment of Diabetic Retinopathy Study chart), slit-lamp examination, intraocular pressure (IOP) measurement (by pneumotonometry), and AS-OCT examination.

### AS-OCT examination

All subjects underwent CASIA2 AS-OCT (Tomey Corp., Nagoya, Japan) imaging in both eyes. The device uses a 1310 nm swept-source laser with a scan speed of 50.000 A-scans per second, freezing 16 scans from 16 different radial angles to produce a three-dimensional analyses of the results [26]. The resolution of the device is 10 μm axial × 30 μm transverse. Two consecutive “Corneal Map” scans were acquired in all included eyes by an experienced operator. All examinations were performed under non-mydriatic conditions in dim room illumination.

### IOL analyses

**Fig. 1:**
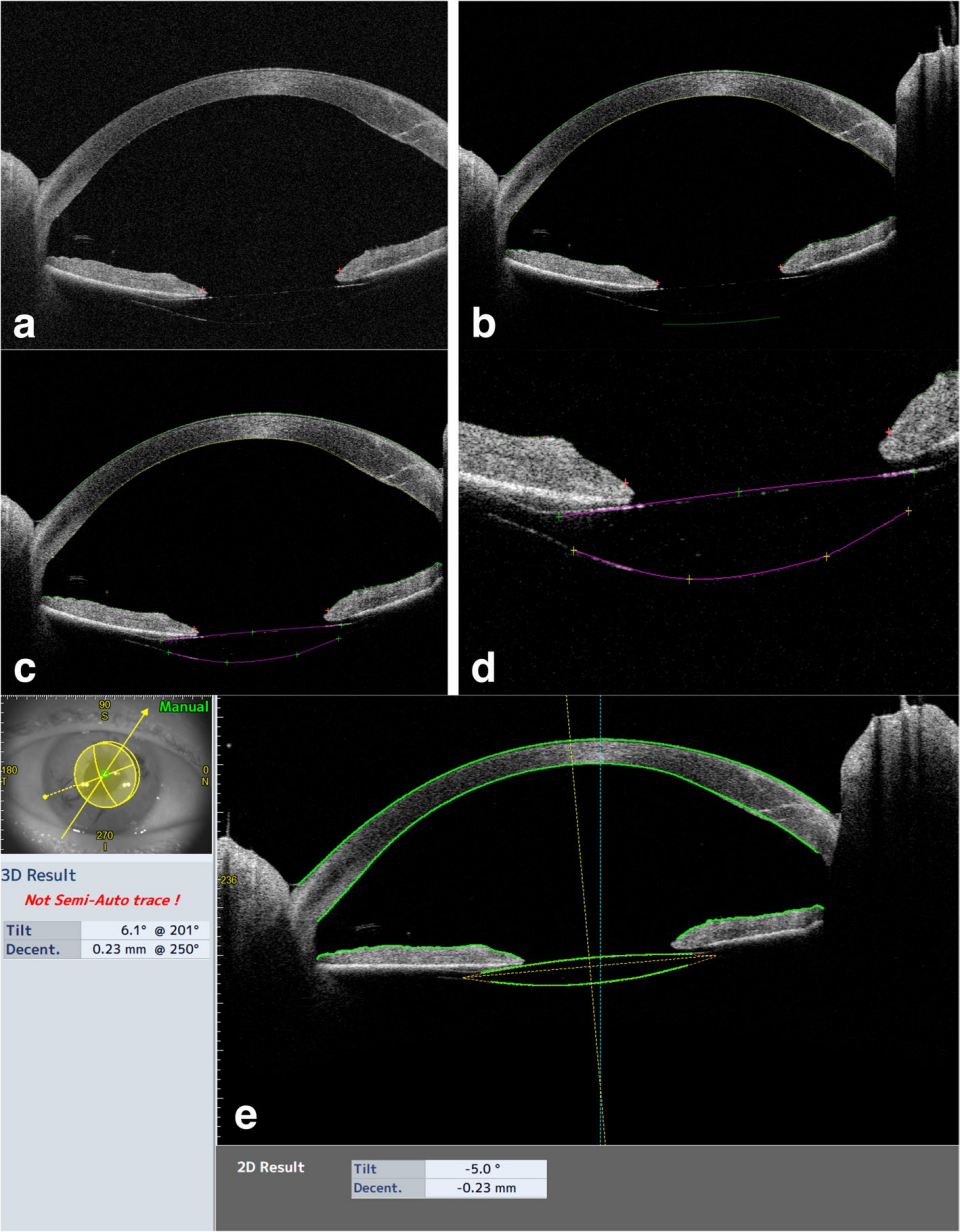
Image **a** shows the CASIA2 posterior IC-IOL OCT scan. Image **b** represents the result of the automatic scan by the CASIA2 software. The cornea and iris profile are perfectly recognized, but the IC-IOL profile is not correct. As shown in image **c**, a manual delineation of the posterior and anterior faces of the IC-IOL is performed to allow for CASIA2 software IOL recognition; the anterior face of the IC-IOL is manually modified from flat to slightly convex (image **d**), allowing for precise recognition of the IC-IOL (image **e**)

**Fig. 2:**
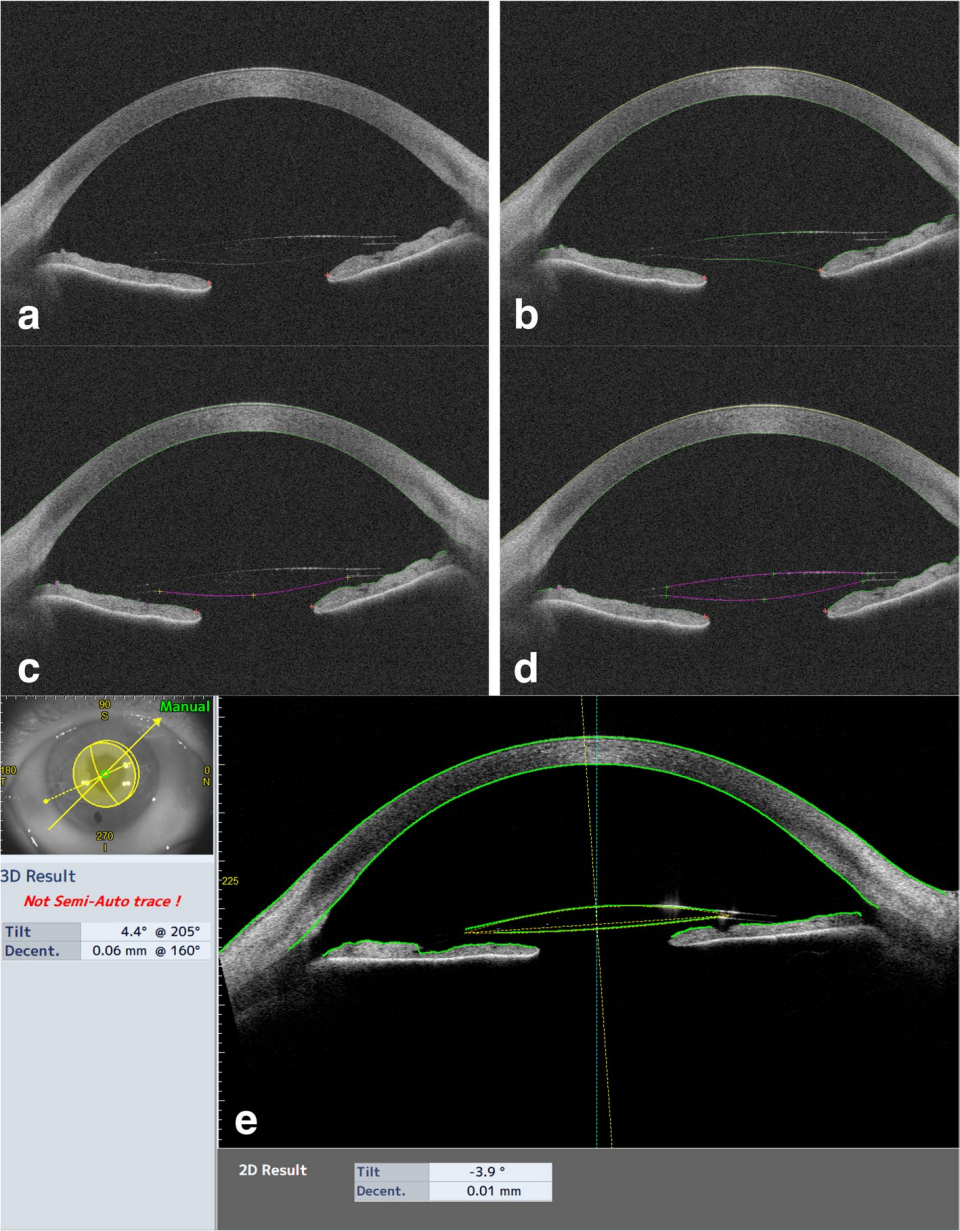
Image **a** shows the CASIA2 anterior IC-IOL scan. Image **b** represents the results of the automatic scan by the CASIA2 software; the cornea and iris profile are perfectly recognized, but the IC-IOL profile is not correct. As shown in images **c** and **d,** a manual delineation of the posterior and anterior faces of the IC-IOL is performed to allow for CASIA2 software IOL recognition (image **e**)

The on-board analyses software (Version 3E.2) was applied for IOL analyses. The “Lens Analysis” function of the software features a tool (“Auto Trace”) that automatically fits a trace line to the anterior and posterior lens profiles in all 16 radial scans and returns a measure of lens tilt and decentration with respect to a visual line. Decentration is defined as the vertical distance from lens center to vertex normal, while the tilt is defined as the angle of the lens axis against vertex normal [27]. The vertex normal is defined as the line that connects the fixation point to the vertex of the corneal topographic map. The decentration is expressed as decentration amount in mm and as the direction in degrees, while tilt is expressed as tilt amount in degrees and direction in degrees. The “Auto Trace” can automatically recognize crystalline lenses and conventional in-the-bag (IB) IOLs but does not properly recognize IC-IOLs. However, the software allowed for a semi-manual tracing of anterior and posterior IC-IOL profiles using the “Semi-Auto Trace” function. Each of the 16 radial profiles obtained using the “Semi-Auto Trace” was carefully checked by an experienced operator (PM) and further manual correction was applied when required. Brightness and contrast of the images were adjusted to improve IOL visualization during the manual delineation, which was performed at 100% zoom to ensure accuracy. The procedure is detailed step by step in Figs. 1 and 2 for PC and AC placement, respectively. Once the correct profile was traced in each of the 16 radial scans, the software could analyze the IC-IOL, providing tilt and decentration values.

Furthermore, anterior chamber depth (ACD) from the corneal endothelium to the anterior IOL edge and pupillary diameter (PD) were automatically measured using the topography function.

### Surgical technique

All eyes were implanted with an Artisan® IC-IOL, which is a rigid poly (methyl methacrylate) (PMMA) IOL with 8.5 mm length, 1.04 mm maximum height, and 5.4 mm optical zone width. The IOL power was calculated with the IOLMASTER® 500 biometer (Carl Zeiss Meditec, Germany) by applying the SRK/T formula and targeting emmetropia in all eyes. An A-constant of 115.0, as per the manufacturer’s recommendations, was used for anterior implantation, while an A-constant of 116.5 was used for retropupillary fixation. The type of anesthesia was either general or peribulbar. Whenever indicated, the procedure was combined with removal of the preexisting lens/IOL or with a pars plana vitrectomy. The same two surgeons (SAT and PM) were involved, both performing anterior and posterior placement. The choice was chiefly driven by the patient’s iris condition (i.e. the presence or not of atrophic areas, iris sutures, wide iridectomy etc.). Two side ports were performed at the nasal and temporal meridian, and anterior vitrectomy was performed to clean the anterior chamber from any vitreous remnants. Then miosis was obtained by intracameral carbachol injection, and cohesive viscoelastic was injected. A superior limbal corneal incision 5.5 mm wide was fashioned and the IOL inserted into the AC with the vault facing up (anterior iris enclavation) or down (posterior iris enclavation). The surgeons rotated the IOL haptics to position them at the 3 and 9 o’clock meridians. A special Artisan lens forceps was then used to hold the IOL’s optic. For AC implantation, a midperiphery portion of the iris was enclavated between the claw-shaped haptics with the dedicated special microspatula introduced through the respective side port. For posterior chamber retropupillary fixation, one haptic of the IOL was positioned behind the iris and the iris tissue was enclavated between the claw haptics using a smooth microspatula. The IOL body was then transferred into the posterior chamber, and the other haptic fixed subsequently. Only in cases of AC implantation, a superior peripheral iridectomy was performed. Interrupted 10–0 non-absorbable nylon sutures were used to close the corneal incision, which were removed 6 weeks after surgery.

### Statistical analyses

For continuous or categorical variables (e.g., age, laterality, ACD, PD), the mean with standard deviation (SD) or the rate (%) was calculated and the Student’s *t* or the Fisher’s exact test were used to compare eyes with the implant between the PC (PC group) and AC (AC group). For linear and angular values associated with decentration and tilt, the median and interquartile ranges (IQR) were calculated. The Bland-Altman method was used to evaluate the agreement between measurements in the two consecutive images of each study eye. Statistical analyses were performed using commercial software (SPSS version 25.0; IBM, Armonk, NY, USA); *P* < 0.05 was considered significant.

## Results

A total of 21 subjects (8 men and 13 women; mean age 77 ± 8 years) fulfilled the study criteria. Each participant had the IC-IOL in one eye (21 eyes; 9 OD and 12 OS); in 14 eyes, the implant was placed in the PC and in 7 eyes the implant was placed in the AC. The average time lasted from surgery was 21 ± 9 months. In Table 1, the characteristics of the two groups are detailed and compared; indications for IC-IOL implantation are summarized in Table 2. The only parameter showing a significant difference between the groups was the ACD (lower in the AC-group, *P* < 0.01). AS-OCT scans of nine fellow eyes with regular IB pseudophakia were also acquired using the same instrument.

**Table 1:** Demographic and clinical characteristics of participants (mean ± SD)

	PC- GROUP (***N*** = 14)	AC-GROUP (***N*** = 7)	***p*** value
**Age (years)**	76 ± 8	78 ± 7	0.58
**Spherical equivalent (D)**	−0.9 ± 1.9	−0.5 ± 1.0	0.61
**BCVA (LogMAR)**	0.37 ± 0.4	0.24 ± 0.2	0.37
**Eye implanted (OD/OS)**	6/8	3/4	
**IOP (mmHg)**	15.4 ± 4.6	14.5 ± 3.4	0.69
**Anterior chamber depth (mm)**	4.1 ± 0.4	2.7 ± 0.2	< 0.01^*^
**Pupillary diameter (mm)**	3.9 ± 0.7	4.0 ± 1.0	0.97

*PC* Posterior chamber implant, *AC* Anterior chamber implant, *D* Diopters, *BCVA* Best corrected visual acuity, *IOP* Intraocular pressure

**Table 2:** Clinical causes for Iris-claw IOL implant

	PC- GROUP (***N*** = 14)	AC-GROUP (***N*** = 7)
**Ocular trauma**	1	2
**PEX syndrome causing lens/IOL luxation**	4	3
**Capsular rupture**	1	1
**Other**	8	1

*PC* Posterior chamber implant, *AC* Anterior chamber implant, *PEX* Pseudoexfoliation syndrome

### Tilt and decentration

**Fig. 3:**
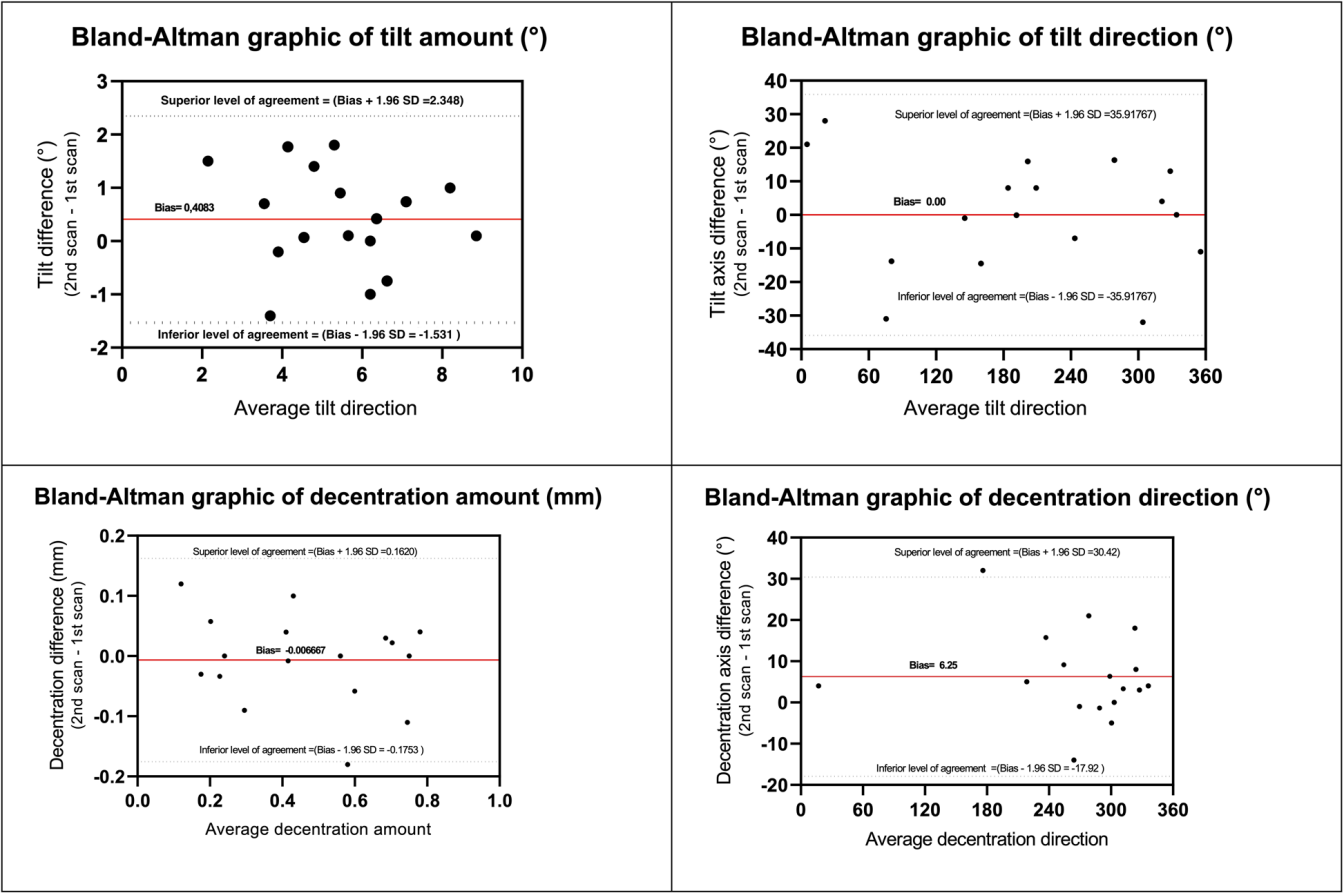
The Bland-Altman analyses of repeatibility for both tilt and decentration

**Fig. 4:**
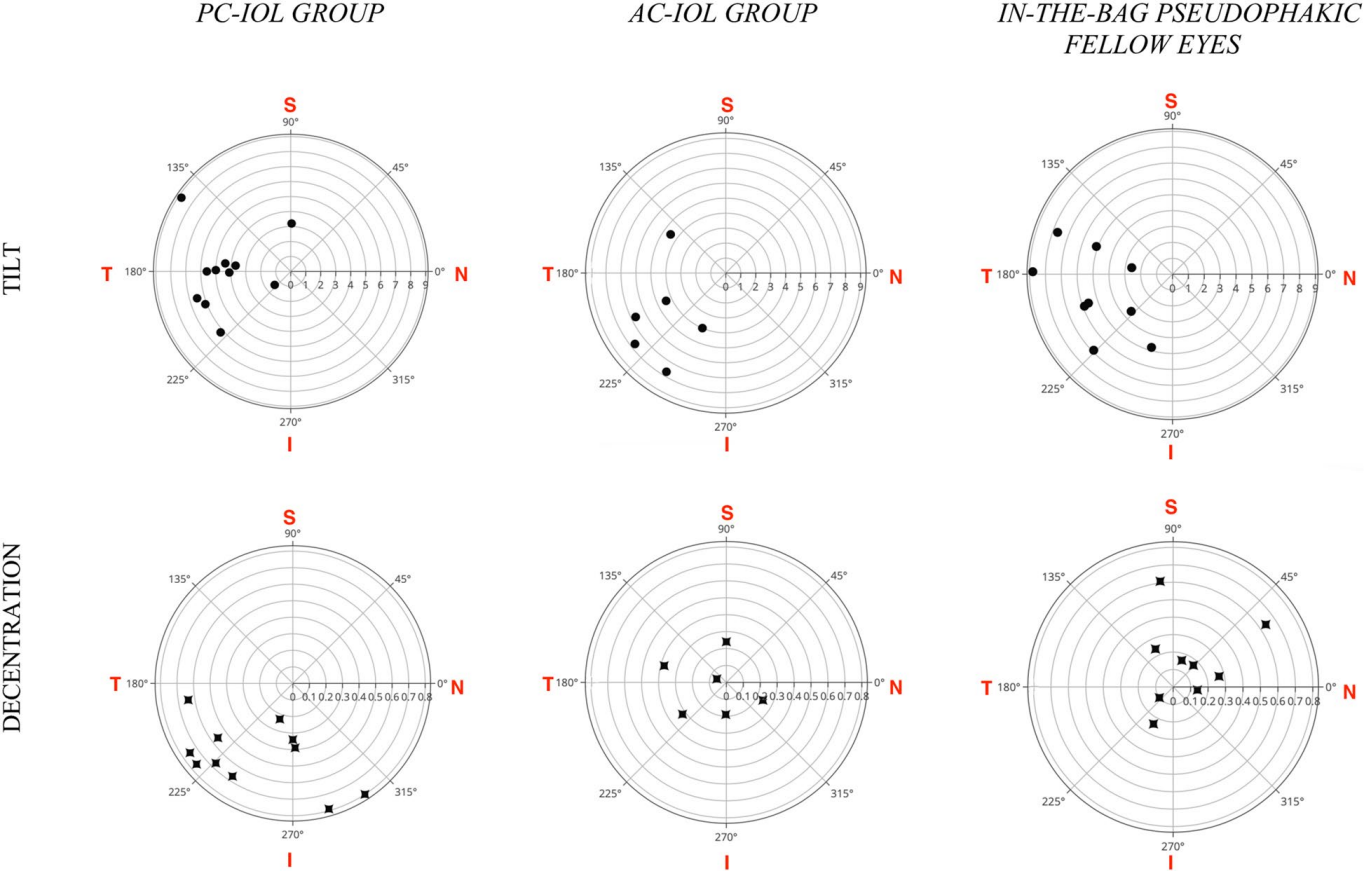
Polar charts representing IC-IOLs tilting (upper part, circle dots) and decentration (lower part, squared dots) of both PC and AC-groups. The amount of tilting is represented in degrees (°), while the amount of decentration is represented in millimeters (mm). IB pseudophakic eyes are shown as a comparison. Left eye and right eye data were merged in a single plot by converting into right eye data for comparison

The semi-manual procedure allowed tilt and decentration measurements to be obtained in 17/21 IC-IOLs, while the software did not calculate these parameters in four patients because of severe iris distortion/IC-IOLs displacement. Repeatability of IC-IOLs tilt and decentration measurements was tested from two scans consecutively acquired in each eye. The Bland-Altman analyses of both tilt and decentration showed that all data (except one axis direction in the AC-group) fell within the 95% limits of agreement (respective plots are shown in Fig. 3). Decentration and tilt values, both expressed as amount and axis direction, are reported in Table 3; the values are detailed for single study eye and median (with IQR) in the PC/AC group and in the OD/OS subgroups. The median decentration was 0.67 mm (0.39–0.76 mm) and 0.24 mm (0.15–0.33 mm) in the PC group and AC group, respectively. The median tilt was 5.0° (3.7°-6.2°) and 5.6° (4.3°-7.7°) in the PC group and AC group, respectively. In the nine IB pseudophakic eyes, the median decentration was 0.24 mm (0.15–0.44), while the median tilt was 5.6° (4.2°-7.3°). Given the limited sample size, no statistical analyses other than pure descriptive were performed for comparison. Figure 4 shows the distribution of tilt and decentration in all study eyes. The three-dimensional placement of the implants is represented through bidimensional polar chart plots virtually centered on the pupil. In all groups, the tilt was towards the 180 temporal degrees. The decentration was scattered in both the AC and IB pseudophakia groups, whereas the decentration of PC IC-IOLs was oriented towards the inferior 180° in all cases and towards the inferior-temporal quadrant in 8/11 (73%) eyes.

**Table 3:** Tilt and decentration values for posterior and anterior chamber iris-claw implants

***IMPLANT IC LOCATION***	***PATIENT (N°)***	***EYE IMPLANTED***	***AMOUNT OF TILTING (°)***	***TILTING DIRECTION (°)***	***AMOUNT OF DECENTRATION (mm)***	***DECENTRATION DIRECTION (°)***
***POSTERIOR***	1	OD	6.5	196	0.79	286
	2	OD	6.1	201	0.23	250
	3	OD	3.7	174	0.64	189
	4	OD	8.8	146	0.80	303
	5	OD	5.6	180	0.56	216
	6	OS	1.4	220	0.67	237
	7	OS	6.2	221	0.34	270
	8	OS	3.2	89	0.76	220
	9	OS	5.0	179	0.75	214
	10	OS	4.4	173	0.67	226
	11	OS	4.1	181	0.39	272
	*MEDIAN OD (IQR)*		*6.1 (4.65–7.65)*		*0.64 (0.39–0.79)*	
	*MEDIAN OS (IQR)*		*4.25 (2.75–5.3)*		*0.67 (0.38–0.75)*	
	*MEDIAN OU (IQR)*		*5.0 (3.7–6.2)*		*0.67 (0.39–0.76)*	
***ANTERIOR***	12	OD	4.4	205	0.06	160
	13	OD	4.5	145	0.24	90
	14	OD	4.0	247	0.24	334
	15	OS	6.7	206	0.19	269
	16	OS	7.7	218	0.38	165
	17	OS	7.7	239	0.32	216
	*MEDIAN OD (IQR)*		*4.4 (4.0–4.5)*		*0.24 (0.06–0.24)*	
	*MEDIAN OS (IQR)*		*7.7 (6.7–7.7)*		*0.32 (0.19–0.38)*	
	*MEDIAN OU (IQR)*		*5.6 (4.3–7.7)*		*0.24 (0.15–0.33)*	

*IQR* Interquartile ranges

## Discussion

We found that the semi-manual tracing function of the CASIA2 AS-OCT can be used to measure tilt and decentration of IC-IOLs, providing repeatable and affordable measurements. In recent years, the automated measurement of tilt and decentration of conventional IOLs as a reference to the corneal topographic axis has become feasible using a second generation AS-OCT. Along with minimizing possible biases related to the pupil shape, it allows for clearer imaging of the cornea and IOL due to the improved scan rate, scan depth, scan density, and a three-dimensional analysis of the results. In this study, we measured tilt and decentration of a specific type of IOL, i.e., the IC-IOL placed in the AC or in the PC.

In our pilot setting, a suitable assessment of the study parameters was achieved in 17/21 of the examined IC-IOLs using a semi-manual image tracking procedure, which showed excellent levels of repeatability. A recent report on the decentration and tilt measurement applied to conventional IB IOLs using the same instrument indicated that a manual adjustment of the IOL marks was performed in all cases to ensure accuracy of the measurements [28]. However, in our cohort the automated measurement of regular IB IOLs was always achieved when the examination involved regular pseudophakic eyes. In addition to difficulties in the identification of the rear IOL surface through an undilated and often irregular pupil, we explain the failure of the full-automated tracing system by the non-predisposition of the algorithm software to recognize a surface as an IOL profile if not convex, as confirmed by the CASIA2 producer. For clawed retropupillary, the IC-IOL surfaces are both concave towards the cornea.

For the decentration, the median amount in the PC-group (0.67 mm [0.39–0.76 mm]) was higher than in the AC-group (0.24 mm [0.15–0.33 mm]). Although no comparative statistics were made because of the limited size of the two samples, decentration in the AC-group was similar to that measured in the regular pseudophakic eyes and lower than in the PC-group. A further descriptive difference between the PC- and AC-group was observed regarding the direction of the decentration, as shown in the lower part of Fig. 4. The PC-group showed a decentration axis oriented towards the inferior hemi-circumference of the eye, while the decentration was scattered in the AC-group. These differences may correlate with the indirect visualization of the IC-IOL haptics during the posterior IC-IOL fixation. Until now, calculation of the decentration of IC-IOLs implanted in the PC has been addressed by referring to the postoperative refractive results and pupil stretching/distortion. Due to these indirect elements of evaluation, decentration may not have been considered a relevant finding [8]. Being able to obtain an accurate measurement of this parameter could affect the surgical procedure by increasing the precision of the IC-IOL placement [29]. Concerning evaluation of the amount and direction of the tilt, these parameters showed a remarkable similarity in all groups. The IQR values for both the PC- and AC-group ranged between 3.7° and 7.7° with an absolute prevalence in the temporal direction (16/17 eyes). This was similar to that obtained in our pseudophakic eyes and reported by two other studies referred to as regular IB IOL groups [26, 28].

We acknowledge limitations to our study, including the small sample size that limited comparative statistical analyses and the lack of optical aberration data that could be correlated with the measured parameters.

## Conclusion

Using the CASIA2 AS-OCT we measured tilt and decentration of IC-IOLs implanted in the AC or PC of aphakic eyes using a semi-manual image tracking procedure. Measurements had good repeatability and showed a decentration towards the inferior 180° for the PC implants. AC implants showed decentration and tilt distribution closer to that observed in regular in-the-bag pseudophakic fellow eyes. Larger studies are required to confirm our results.

## Data Availability

All data and material are available from the corresponding author.

## Abbreviations

AC: Anterior chamber
ACD: Anterior chamber depth
AS-OCT: Anterior segment optical coherence tomography
BCVA: Best-corrected visual acuity
IB: In-the-bag
IC-IOLs: Iris-claw intraocular lenses
IOL: Intraocular lens
IQR: Interquantile range
PC: Posterior chamber
PD: Pupillary diameter
PMMA: Poly (methyl methacrylate)
UBM: Ultrasound biomicroscopy
